# Between Scylla and Charybdis

**DOI:** 10.1007/s12471-019-1290-x

**Published:** 2019-05-21

**Authors:** H. J. te Kolste, G. J. Kimman, T. Germans, S. A. J. Timmer

**Affiliations:** 1 Location VU Medical Center, Amsterdam University Medical Center, Amsterdam, The Netherlands; 2 North-West Hospital Group, Alkmaar, The Netherlands

## Answer

**Fig. 1:**
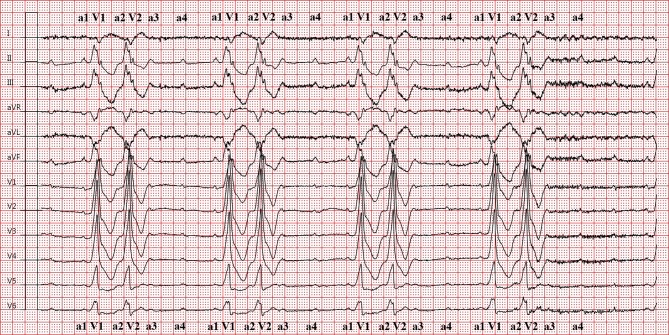
Electrocardiogram on admission after syncope

**Fig. 2:**
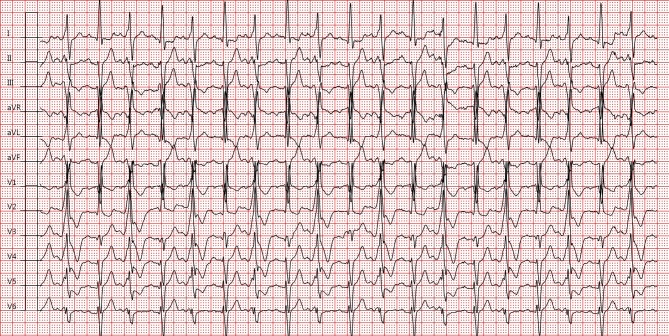
Electrocardiogram after isoprenaline

The electrocardiogram (Fig. 1) shows a sinus tachycardia with third-degree atrioventricular block. Two very broad QRS complexes with identical morphology occur with regular intervals (V1 and V2). These complexes are caused by antegrade conduction of consecutive sinus beats a1 and a2 entirely over the accessory pathway, resulting in a fully pre-excited QRS complex. The following sinus beats (a3 and a4) are not conducted due to refractoriness of the accessory pathway and either retrograde invasion of the left posterior fascicle (LPF) by the previous conducted sinus beats or consistent antegrade atrioventricular block. The possibility of an intermittent antidromic circus movement tachycardia seemed less likely, due to strictly regular p‑p intervals. Atrioventricular conduction was improved after administration of isoprenaline (Fig. 2), and resulted in 1:1 conduction of sinus beats alternating between the His-Purkinje system and accessory pathway, the latter QRS complex being slightly narrower than before as a result of fusion with native conduction. Obviously, flecainide was discontinued and the patient was planned for definitive pacemaker implantation prior to electrophysiology studies.

In patients with Wolff-Parkinson-White syndrome, flecainide mainly prolongs the refractoriness of the accessory pathway and is used to prevent atrial tachyarrhythmias (e.g. atrial fibrillation) from rapidly conducting in the presence of a ‘malignant’ pathway [1]. However, flecainide may lead to third-degree atrioventricular block, especially in patients with advanced conduction disease. Fortunately for this patient, asystole was presumably averted by preserved atrioventricular conduction over a ‘life-saving’ accessory pathway. We must emphasise that caution is advised prescribing flecainide to patients with pre-existent chronic bifascicular block.

## References

[1] Andrikopoulos GK, Pastromas S, Tzeis S (2015). Flecainide: Current status and perspectives in arrhythmia management. World J Cardiol.

